# Essential leadership concepts and models for graduate and professional school learners

**DOI:** 10.1002/yd.20531

**Published:** 2022-12-24

**Authors:** Ralph A. Gigliotti, Sara E. Spear

**Affiliations:** ^1^ Center for Organizational Leadership Rutgers University New Jersey USA

## Abstract

This article explores the importance of providing leadership development opportunities for graduate and professional students, in addition to highlighting approaches for building leadership capacity among these students. The article concludes with a snapshot of leadership development offerings for graduate and professional audiences sponsored by institutions (curricular and co‐curricular), corporations, and professional development associations.

## INTRODUCTION

Graduate and professional education curricula provide discipline‐specific content for students to study and apply in their chosen fields. However, the immense challenges of the historical moment, the rapid speed of change, and the competencies required for success in the knowledge economy often demand more than the knowledge and skills that are unique to one's discipline of study. This is explored by the Kaufman and Stedman article in this issue. Although subject matter expertise remains undeniably critical—medical doctors must understand physical anatomy, lawyers require knowledge of regulations, statutes, and laws, and accountants need to be proficient in the review of balance sheets and financial statements—but one's effectiveness in such roles also requires cross‐cutting skills that extend beyond one's discipline. Lubker and Petrusa in their article within this issue share two examples of co‐curricular programs designed to enhance graduate students’ leadership capacities. Colleges and universities have a responsibility to equip their graduates with the broad range of competencies necessary for engaging in the contemporary workplace.

As found in Werner's article in this issue, while there has been extensive attention paid to scholarship and programming related to undergraduate leadership education and development, the focus remains underemphasized among graduate and professional students. As a result, these students may graduate and enter the workforce lacking the necessary skills and experience to move into roles and take on responsibilities where they can effectively enact leadership, which could prove problematic for the student and the teams, organizations, and sectors that are beneficiaries of one's leadership. As opposed to viewing leadership development as detracting from the essential curricula and primary goals of one's academic program, the need exists for a more purposeful and intentional integration of leadership concepts, strategies, and practices into the curricular and co‐curricular experience for graduate and professional education. With this need in mind, this article explores the importance of providing leadership development opportunities for graduate and professional students, in addition to highlighting approaches for building leadership capacity among these students.

## A CLOSER EXAMINATION OF LEADERSHIP DEVELOPMENT

Leadership has been studied for centuries and the evolution of theories and research have offered broadened views of the subject, ranging from initial studies of the innate traits of individual leaders to more expansive considerations of followers and contexts in contributing to the dynamics of leadership as an interactive, communicative, and co‐constructed process of social influence (Fairhurst & Connaughton, 2014; Ruben & Gigliotti, 2016; Ruben et al., 2021). Contemporary scholarship tends to emphasize the multidirectional interactions and intersections that lie at the nexus of leaders, followers, and their contexts (Kellerman, 2012). The competencies needed to effectively engage in leadership can be developed using a variety of methods, including the use of formal leadership development programs that either accompany, parallel, or become integrated into learning and development initiatives. Such initiatives aim to expand, enhance, and enrich leadership capacity among participants so they can engage more effectively in the process and practice of social influence in pursuit of their individual and collective goals.

### Benefits to the graduate and professional student

Providing graduate and professional students with opportunities for enhanced leadership development and education can significantly benefit the individual learner, as well as their academic discipline and the various communities within which they belong. These positive outcomes assume that students take advantage of these opportunities and demonstrate engagement, commitment, and persistence in the development process (Conger, 1992; Kwok et al., 2020).

### Socialization

The socialization of graduate students unfolds as they interact with faculty, classmates, and colleagues from across the discipline, and as they deepen their understanding of the culture, norms, and expectations of their academic community. Socialization, which occurs across many levels, has been found to be closely linked with persistence and degree attainment for doctoral students (Tinto, 1993). The locus of doctoral education lies within the department, rather than the institution (Golde, 2005), and socialization processes are often cultivated within one's department and forged through relationships with one's primary advisor. Through the process of socialization, students can develop a heightened sense of belonging in the community, forming relationships and connections within one's department, institution, and discipline. For some graduate and professional programs, socialization efforts may be more appropriately expanded to consider the cohort or school context.

In addition to introducing students to the culture of the department or school, students are, by extension, simultaneously immersed in their broader discipline. Therefore, leadership development can enhance the professional identity formation process where students begin to make meaning of their academic or professional community and define themselves in relationship to their desired profession—an ongoing process that initially takes shape during their time as a student (Wong & Trollope‐Kumar, 2014). For example, in medical school, students complete clinical rotations where they interact with patients, experience daily healthcare operations, and begin to develop their professional identities as healthcare providers (Wong & Trollope‐Kumar, 2014). Leadership development efforts can play an active role in this process and equip students in cultivating leadership identities alongside their professional identities.

In addition to departmental and disciplinary socialization, graduate and professional education can serve as a laboratory for understanding and engaging with interdisciplinary practice. Valued by both academia and industry, interdisciplinary and transdisciplinary works are often reflected in team research, innovative course curricula, and new centers or institutes that span across disciplinary boundaries. Despite calls for the expansion of interdisciplinary and transdisciplinary efforts, doctoral students continue to be prepared in traditional single‐discipline programs with limited opportunities for interdisciplinary interactions. Two examples of graduate student leadership development programs that allow for increased interdisciplinary connections will be discussed in a later section of this article: the Pre‐Doctoral Leadership Development Academy at Rutgers University (Gigliotti et al., 2016) and Doctoral Student Leadership Institute at the University of South Florida (Terry & Liller, 2014). The University of Notre Dame's LASER program described in Lubker and Petrusa's article within this issue is another wonderful example.

With structured opportunities for socialization and facilitated interactions across disciplines, students can assimilate into the wider campus community. Thus, leadership development offerings play a role in preparing students who may take on leadership positions and responsibilities in organizations and initiatives that impact the graduate student experience. Serving in these campus roles, students can develop confidence and competence in pursuing leadership opportunities throughout their professional careers. Furthermore, formal and informal leadership development opportunities at the graduate level may introduce and inculcate a set of expectations and values that are most desired among future leaders.

### Transferrable skills

For graduate and professional students who pursue careers in academia, success is often determined by one's individual achievements in research, teaching, and for some, clinical practice—and these remain areas of greatest emphasis in the curriculum. However, as these individuals progress in their careers, they often acquire administrative roles or engage in projects that may require a different set of competencies. This transition from faculty member to administrator necessitates a shift from the individual to the collective, specialist to generalist, autonomy to accountability, and disciplinary to institutional loyalty (Gmelch, 2015; Raines & Alberg, 2003; Ruben et al., 2021). Martin explores many of these issues faced by administrators and faculty members within his article in this issue.

For many graduate and professional programs, leadership development remains a peripheral goal, and there is an opportunity for graduate programs to further prepare students for their potential transition to future administrative roles. Just as medical knowledge alone does not guarantee high‐quality patient care—personalized care that requires proficiency in communication, critical thinking, ethical decision‐making, and professionalism—leadership extends beyond the specific knowledge or skills of any one specific subject area.

Some models highlight a selection of competencies that are most directly associated with leadership effectiveness. According to the National Association of Colleges and Employers (NACE), the list of competencies employers expect among college graduate applicants include teamwork, problem‐solving, analytical, and both verbal and written communication skills (Gray, 2021). These competencies are the same ones often emphasized in leadership development programs. In a review of healthcare‐oriented student leadership development programs, for example, the topics of effective communication and effective teamwork are most frequently addressed, in addition to a focus on leading change, strategic planning, and organizational culture—topics which transcend the specialized and technical knowledge and skills that are critical to one's graduate and professional education (Spear, 2020). In addition to equipping participants with cross‐cutting leadership competencies, such opportunities prove especially beneficial for women, people of color, and first‐generation students who may be underrepresented in their fields and may not come from backgrounds of privilege that would have afforded opportunities for distinguishing oneself from other candidates.

### Preparation for an uncertain future

Those engaged in leadership moving forward will need to address a wide array of environmental, social, and economic challenges, including, but not limited to climate change, poverty, immigration, and access to healthcare. As one example, the healthcare sector remains in a state of rapid evolution and change, and modern challenges such as population health issues presented by the COVID‐19 pandemic require physician leaders and other healthcare professionals who can provide high quality care and still adapt and exert positive influence in these settings (Mokshagundam et al., 2019; Neeley et al., 2017). In support of team‐based care models, medical education has widely promoted interprofessional education (IPE) as a strategy to adequately prepare students to work on teams of health professionals. IPE promotes interdisciplinary communication, collaboration, and teamwork, all of which are essential competencies for leaders (Moskhagundam et al., 2019). Regardless of one's career trajectory, the challenges of tomorrow require a dedicated and intentional focus on leadership development in today's graduate and professional education programs.

## LEADERSHIP DEVELOPMENT LANDSCAPE

### Approaches to leadership development

Existing program models are quite varied including formal coursework or educational seminars and co‐curricular experiential opportunities such as student organizations or service‐learning. Some approaches can be differentiated by *leader* and *leadership* development, where Day (2001) described the former as focusing on the intrapersonal dimension and the latter on interpersonal and collective dimensions, and where the ideal intervention takes account of both dimensions. Some programs focus on specific leadership theories, such as transformational or servant leadership, or utilize a model like Kolb's (1984) experiential learning model or the social change model of leadership development (Astin & Astin, 1996). Others, like the Executive Leadership and Organizational Change program at Northern Kentucky University (Rhee & Sigler, 2020), use emotional intelligence (Goleman, 2005) as a central pillar of their work. The Council for the Advancement of Standards (CAS) in Higher Education recommends the use of multiple leadership theories in informing program design rather than exclusively employing one individual theory (as cited in Deal & Yarborough, 2020).

### Components of existing programs

Conger (1992) described four important dimensions of leadership education as involving conceptual understanding, skill building, personal growth, and feedback. Effective programs typically address each of these areas, though not necessarily equally, and use a variety of instructional strategies (Allen & Hartman, 2008). For example, lectures and guest speakers can be used for conceptual understanding, simulations and games for skill‐building, and reflection for personal growth. Feedback, the fourth dimension of Conger's (1992) model, may include various evaluative instruments, executive coaching, and mentoring (Allen & Hartman, 2008; Lucas et al., 2018). The core components of leadership development programs vary based on program goals and focal audience (Gigliotti, 2017), and effective leadership development programs often employ multiple instructional and learning methods.

Experience, reflection, and social interactions are all sources of learning within leadership development. Institutions and programs can consider the developmental experiences (DeRue & Wellman, 2009) they provide to help students hone their leadership skills. In addition, leadership development programs can tap into each learner's implicit leadership theory that explains one's view of effective leadership based on individual experiences (Souba & Souba, 2018). Programs that acknowledge individual views of leadership can help facilitate meaning‐making through reflective journaling or facilitated dialogue where learners interact, contributing diverse ideas and experiences, and co‐constructing new knowledge with one another in the process (Mezirow, 1990, 1997). Mentorship or coaching are other strategies that promote social interaction and they are particularly important for women and minorities who may be underrepresented in their fields or in the leadership ranks (Mokshagundam et al., 2019). The most effective initiatives are likely those that incorporate various components for leadership development.

## SNAPSHOT OF DEVELOPMENTAL APPROACHES

Since leadership development can take many forms, one way to categorize approaches to graduate‐level leadership education is through a consideration of the multiple contexts for programming (Guthrie & Jenkins, 2018). Leadership programs may be academic or curricular, co‐curricular, discipline‐specific, interdisciplinary, or rooted in professional development. For the purposes of this article, we provide a snapshot of various leadership development offerings for graduate and professional audiences sponsored by institutions (curricular and co‐curricular), corporations, and professional development associations.

### Curricular

Most graduate‐level programs that focus on leadership are hosted by colleges of education (Terry & Liller, 2014) or are management focused such as those found in MBA programs (Rhee & Sigler, 2020). Additionally, other graduate and professional disciplines integrate a focus on leadership in their formal curriculum. For example, the Elon University School of Law employed a framework for their three‐year leadership development program that involves focusing on the self, leading teams, and leading in the wider profession and community (James, 2011). The program involves team‐building exercises, structured mentorship, self‐assessments, non‐profit and community projects, and the development of individual leadership plans that help students be more intentional about their time in law school and the areas of law which they seek to practice (James, 2011). The non‐profit and community projects are particularly important in cultivating a sense of public service that can be sustained throughout their careers.

Medical education is increasingly integrating leadership development as part of its curricula, alongside traditional basic and clinical sciences (Mokshagundam et al., 2019; Schmidt & Linenberger, 2020). One innovative model has been the Scholarly Excellence, Leadership Experiences, Collaborative Training (SELECT) program, representing a partnership between the University of South Florida (USF) Morsani College of Medicine and Lehigh Valley Health Network (LVHN) in Pennsylvania. In addition to general medical curriculum and learning about patient‐centered care and teamwork in healthcare, all students in the SELECT program receive leadership development organized around emotional intelligence (Monroe & English, 2013). Students spend the first two years at USF, followed by 2 years at LVHN, where they are expected to apply what they have learned, including leadership competencies, during their clinical training (Monroe & English, 2013). The SELECT model showcases the integration of leadership development into the core elements of the curriculum to ensure that students strengthen these competencies alongside their medical knowledge and technical skills.

Schools of pharmacy have identified leadership development as a key component of their accreditation standards, integrating it into orientation and also through the curriculum in individual courses, interprofessional education, and practice requirements, as well as through co‐curricular activities (Janke et al., 2016). However, common barriers for integration include formal curricula that provide little extra time for students to take on another requirement, particularly when other requirements, such as preparing for licensing exams, are top priority (Mokshagundam et al., 2019). When formal coursework cannot be added to the existing curricula, one method has been the development of combined degree programs (e.g., MD/MBA) and certificate programs. The Gordon Engineering Leadership Program at the Massachusetts Institute of Technology (MIT), for example, encourages graduate students to pursue a Graduate Certificate in Technical Leadership which can also be used as a doctoral minor for PhD students (MIT, 2022). The additional coursework on topics related to leadership allow students to explore how these concepts relate to their discipline, although a downside to such efforts may be an extension of the length of one's formal enrollment as a student.

The Center for Creative Learning (CCL) suggests tying leadership development to curriculum whenever possible so they enhance one another, with students making linkages between topics that can then be applied later (Deal & Yarborough, 2020). Such connective experiences that allow for relevant application of learning may be more valuable, and integrating a group project, internship, or service learning into the curriculum is often found to be beneficial to students. For example, in the University of California, Los Angeles (UCLA) PRIME leadership program, medical school students are encouraged to undertake quality improvement projects in their clinical training (Mokshagundam et al., 2019). Similarly, engineering programs frequently include an action project requirement to engage students in design thinking. These applied approaches intentionally integrate leadership development into the curricular experience, as opposed to treating the subject as separate or distinct from the core curriculum.

### Co‐curricular

The curricular components of graduate education are best complemented by co‐curricular activities. Co‐curricluar examples for graduate and professional students are also shared in Lubker and Petrusa within this issue. The Pre‐Doctoral Leadership Development Academy (PLDA) at Rutgers University is one such program that focuses on leadership development for an interdisciplinary group of doctoral students (Gigliotti et al., 2016). With a recognition that faculty are often ill‐prepared to lead complex departments, schools, and institutions, PLDA was launched in 2009 as an “early intervention” program where doctoral students from a variety of disciplines could be introduced to leadership concepts and skills (Gigliotti et al., 2016, p. 46). Participants attend bi‐weekly sessions on a variety of topics related to academic leadership and trends facing the higher education sector. The program “serves as a model for extending doctoral education” and “as a venue for cultivating leaders‐in‐training” alongside their education as scholars (Gigliotti et al., 2016, p. 42). In addition to lectures, case studies, and other pedagogical methods, students engage in a mentoring relationship with a campus leader and design a personalized leadership development plan.

The Doctoral Student Leadership Institute at the University of South Florida is another example of an interdisciplinary program that combines curricular and co‐curricular components. Program fellows, representing a variety of doctoral disciplines, participate in a one‐semester course followed by three semesters where they engage in leadership seminars, shadowing, and other options like service‐learning projects (Terry & Liller, 2014). In this program, students actively participate in a journal or book club where students read and discuss selected leadership readings (Terry & Liller, 2014).

The Distinction in Leadership in Academic Health (DILAH) program at the Rutgers Robert Wood Johnson Medical School is an example of an elective‐based option (RWJMS, 2022). This one‐year program includes a series of classroom meetings on a variety of leadership topics. For students who attend ten of these sessions, completion of the program is added to their transcript as a non‐credit elective and counted similarly to other specialized classes such as Medical Spanish or Trauma, Emergency, and Disaster Response. For students who go on to complete a shadowing experience and capstone project, they receive a special distinction status on their transcript.

Medical students have recommended the creation of more opportunities for formal leadership positions within student‐run clinics and student organizations (Mokshagundam et al., 2019). In addition to these types of roles, other opportunities might include inviting students to sit on various university committees, engage in student governance, or participate in community service projects or teaching assistant opportunities (Neeley et al., 2017). There are also opportunities to practice leadership in interprofessional teams and across institutions. For example, the National CLARION case competition hosted by the University of Minnesota engages interprofessional teams across the country to analyze a case study. Each team represents at least two health disciplines which enables participants to examine healthcare issues and potential solutions using a more comprehensive and interdisciplinary perspective (University of Minnesota, 2022). Co‐curricular experiences such as these can serve as valuable leadership development opportunities for graduate and professional students across disciplines.

### Corporate

For students who graduate and pursue careers in the corporate sector, some industries have long invested in leadership and management development. Fields like human resources, finance, supply chain management, information systems, and engineering areas are areas well‐positioned for these opportunities. Amazon, Johnson and Johnson, and Whirlpool are three corporations that provide rotational internship experiences to graduate students to gain professional and leadership experience through on‐the‐job experience with additional mentorship and career coaching. Deloitte offers a unique approach for leadership development through Deloitte University, “where learning meets leadership” (Deloitte, 2022a, para. 1). Female Master's students are invited to apply to participate in the Deloitte Women's Leadership Launch, a weekend conference event where they attend leadership‐oriented workshops and sessions, build their professional networks, and consider their own strengths and leadership development goals (Deloitte, 2022b). Corporate internships that place an emphasis on leadership development can also prove useful in inculcating the cultural values and professional expectations of the company among participants who will enter the talent pipeline upon graduation.

### Professional associations

Most academic disciplines and professional associations provide networking options through committees and conferences, publications, and resources on a variety of topics including leadership development. Recognizing the value of such resources in leadership development, the Doctoral Student Leadership Institute at the University of South Florida provides program participants with $1000 per semester which is intended to be used for professional conferences, events, or leadership materials (Terry & Liller, 2014).

The American Dental Education Association (ADEA) offers the Student Diversity Leadership Program for dental students, a program targeting “emerging student leaders from diverse backgrounds who have an interest in issues related to diverse communities” (ADEA, 2022, para. 2). The conference‐style event is one method for ensuring that students from underrepresented communities are provided with meaningful leadership opportunities. The American Medical Association (AMA) website hosts the “Medical Student Leadership Learning Series,” where students at AMA‐member institutions can access online modules on a variety of topics related to leadership including effective communication, collaboration, conflict resolution, and managing stress (AMA, 2022). Many associations provide materials and resources that academic institutions can tap into and promote to their students.

## CONCLUSION

In response to many of the changes and challenges across disciplines, systems, and work environments, there is a need for robust and theory‐informed leadership development programs for graduate and professional students. The content provided in this article, along with this larger volume, provide concepts and examples to help inform the design and development of such initiatives. Although there is no one model that will meet all needs, graduate and professional student participants will likely continue to benefit by engaging in ongoing development opportunities that allow these students to step beyond the contours of their disciplines in pursuit of the cross‐cutting domains that are recognized as most critical in the scholarly and professional leadership literature.
